# Supplementary material for: Cannabis Use Is Associated with Pain Severity and Interference Among Cancer Survivors
Source: Integr Med Rep. 2024 Jul 22;3(1):81–91. doi: 10.1089/imr.2024.0001 (PMC11290042; doi:10.1089/imr.2024.0001)
Supplement: Supplemental data [file imr.2024.0001_supplementalfile1.pdf]

Supplemental File 1. Survey

Thank you again for taking this questionnaire. This survey should take about 15 minutes to complete. We ask that you attempt to answer each question honestly and only to skip questions if you absolutely feel you cannot answer. Some of the questions may seem personal, embarrassing, or upsetting. You are free to skip any question that you do not feel comfortable answering.

Benefits and Risks of Cannabis

Study ID ____

When we use the term 'cannabis' we are referring to any marijuana, cannabis concentrates, edibles, lotions, ointments, tinctures, or other products made with cannabis, as well as CBD-only products.

Do you think that there are any benefits related to cannabis use?

◯ Yes
◯ No

What do you believe are the benefits of using cannabis, even if you've never used it? Select all that apply.

☐ Pain management
☐ Relief of stress, anxiety or depression
☐ Relief from neuropathy (numbness or tingling in your hands or feet)
☐ Relief from sweating symptoms (e.g., hot flashes, night sweats)
☐ Improved sleep
☐ Improved nausea/vomiting
☐ Increased appetite
☐ Increased energy
☐ Increased sexual interest or activity
☐ Decreased use of other medications
☐ Decreased use of illicit substances other than cannabis
☐ Managing side effects from cancer treatment
☐ Treatment of cancer
☐ Treatment of another medical condition (i.e., seizures, chronic pain)
☐ Enjoyment or recreation
☐ Other benefits (please specify):
Please specify:

Do you think that there are any risks related to cannabis use?

◯ Yes
◯ No

What do you believe are the risks of using cannabis? Select all that apply.

- ☐ Daytime sleepiness
- ☐ Headache
- ☐ Irritability
- ☐ Impaired memory
- ☐ Difficulty concentrating
- ☐ Dizziness/falls
- ☐ Disruption in sleep
- ☐ Inability to drive
- ☐ Lung damage
- ☐ Addiction to cannabis
- ☐ Increased stress, anxiety, or depression
- ☐ Increased appetite or weight gain
- ☐ Increased use of other prescribed medications
- ☐ Increased use of illicit substances other than cannabis
- ☐ Increased risk of cancer
- ☐ Increased risk of development of other diseases
- ☐ Legal risks
- ☐ Job loss or negative career impacts
- ☐ Negative reactions from family members or friends
- ☐ Other risks (please specify)

  Please specify:

Where would you be most likely to go if you wanted to learn more about cannabis use and cancer? Select all that
apply.

- ☐ Primary care provider
- ☐ Oncologist in charge of your cancer treatment
- ☐ Nurse or physician's assistant involved with your cancer treatment
- ☐ Nutritionist
- ☐ Another cancer patient
- ☐ Friend or family member
- ☐ Cannabis store or dispensary
- ☐ Hospital website
- ☐ Official federal, state, or local government website
- ☐ Pamphlet or handout
- ☐ News or magazine articles
- ☐ Social media or blogs (Facebook, Twitter, etc.)
- ☐ Other (please specify)

  Please specify:

How comfortable would you feel talking with your healthcare providers about cannabis?

◯ Extremely uncomfortable
◯ Somewhat uncomfortable
◯ Somewhat comfortable
◯ Extremely comfortable

Have you discussed using medical cannabis for your cancer symptoms with a healthcare provider?

◯ Yes
◯ No

What type of provider have you talked to about cannabis? Select all that apply.

☐ Primary care provider
☐ Oncologist involved with your cancer treatment
☐ Nurse or physician's assistant involved with your cancer treatment
☐ Pharmacist
☐ Another health care professional

At any time since your cancer diagnosis, has your doctor or another healthcare provider recommended that you use cannabis?

◯ Yes
◯ No

What type of healthcare provider recommended you use cannabis? Select all that apply.

☐ Primary care provider
☐ Oncologist involved with your cancer treatment
☐ Nurse or physician's assistant involved with your cancer treatment
☐ Pharmacist
☐ Another health care professional

Cannabis Use and Cancer

The next questions ask about your use of cannabis before, during, or after your cancer diagnosis or treatment. If you have had more than one cancer diagnosis, please respond based on your most RECENT cancer diagnosis.

Prior to your cancer diagnosis, did you ever, even once, use cannabis for any reason?

◯ Yes
◯ No

Have you used cannabis at any time since your cancer diagnosis?

◯ Yes
◯ No

Have you considered using cannabis since your cancer diagnosis?

◯ Yes
◯ No

What are the reasons you have not used cannabis since your diagnosis? Select all that apply.

☐ Cost is too high
☐ Not covered by my health insurance
☐ My health care providers have not suggested or recommended it
☐ My healthcare providers have recommended against it
☐ I did not think it would be helpful for me
☐ I did not know how to get it
☐ Too many choices or unsure which products are safe and effective
☐ Concern about interaction with other medicines
☐ Concern about side effects
☐ I had a bad experience with cannabis
☐ Concern about legal consequences
☐ Concern about job loss or negative career impacts
☐ Concern about negative reactions from family members or friends
☐ It goes against my personal beliefs
☐ Other (please specify)
Please specify:

Are you currently using cannabis?

◯ Yes
◯ No

When you use cannabis, does it typically contain:

- ◯ Mostly THC with some CBD
- ◯ Mostly CBD with some THC
- ◯ Similar amount of THC and CBD
- ◯ Only CBD
- ◯ Don't Know

In general, where do you obtain your cannabis products (select all that apply)?

- ☐ A medical dispensary
- ☐ A recreational dispensary
- ☐ A pharmacy or grocery store (CBD only products)
- ☐ I don't know

What is your best estimate of the number of days you used cannabis during the past 30 days? ___________________

The next few questions ask about your use of cannabis during and after your cancer treatment.

Did you use cannabis at any time during your cancer treatment?

- ◯ Yes
- ◯ No
- ◯ I haven't started treatment

On average, during your cancer treatment, how often do you or did you use cannabis?

- ◯ More than once a day
- ◯ Daily or almost daily
- ◯ A few times a week
- ◯ A few times a month
- ◯ Once a month or less

Did you use cannabis at any time after the end of your cancer treatment?

- ◯ Yes
- ◯ No

On average, after your cancer treatment, how often do you or did you use cannabis?

- ◯ More than once a day
- ◯ Once a day or almost every day
- ◯ A few times a week
- ◯ A few times a month
- ◯ Once a month or less
- ◯ Only tried it once or twice

Who is the main person that gives you instructions on how to use cannabis and how much to take?

- ◯ Primary care provider
- ◯ Oncologist involved with your cancer treatment
- ◯ Nurse or physician's assistant involved with your cancer treatment
- ◯ Pharmacist
- ◯ Nutritionist or dietician
- ◯ Cannabis store or dispensary worker
- ◯ Unlicensed cannabis dealer or seller
- ◯ Another cancer patient
- ◯ Friend or family member
- ◯ Other (please specify)
- ◯ No one gives me instructions

Please specify the person who gives you instructions: ____________________________

At any time since your cancer diagnosis, have you used cannabis in the following ways? Select all that apply.

- ☐ Smoking (for example, in a joint, bong, pipe, or blunt)
- ☐ Eating (for example, in brownies, cakes, cookies, or candy)
- ☐ Drinking (for example, in tea, cola, or alcohol)
- ☐ Taking by mouth such as pills, tinctures, or sublingually (under the tongue)
- ☐ Vaping or vaporizing (for example, in an e-cigarette-like vaporizer or other vaping device)
- ☐ Dabbing (for example, using waxes or concentrates)
- ☐ Applying topically (for example, in a lotion or cream)
- ☐ Other (please specify)

Please specify:

Which one of the following ways do you, or did you, use cannabis most often since your cancer diagnosis?

- ◯ Smoking (for example, in a joint, bong, pipe, or blunt)
- ◯ Eating (for example, in brownies, cakes, cookies, or candy)
- ◯ Drinking (for example, in tea, cola, or alcohol)
- ◯ Taking by mouth such as pills, tinctures, or sublingually (under the tongue)
- ◯ Vaping or vaporizing (for example, in an e-cigarette-like vaporizer or other vaping device)
- ◯ Dabbing (for example, using waxes or concentrates)
- ◯ Applying topically (for example, in a lotion or cream)
- ◯ Other (please specify)
  Please specify:

What were your reasons for using cannabis after your cancer diagnosis? Select all that apply.

- ☐ Pain
- ☐ Mood changes, stress, anxiety, or depression
- ☐ Neuropathy (numbness or tingling)
- ☐ Difficulty sleeping
- ☐ Difficulty concentrating
- ☐ Skin problems
- ☐ Sweating symptoms (e.g., hot flashes, night sweats)
- ☐ Digestive problems (e.g., nausea, vomiting, diarrhea, constipation)
- ☐ Lack of appetite
- ☐ Lack of energy
- ☐ Lack of sexual interest of activity
- ☐ Used as treatment for cancer
- ☐ Used recreationally or for enjoyment
- ☐ Used for a cancer symptom or cancer treatment side effect not listed here (please specify)
  Please specify symptom: ____
- ☐ Other reason (please specify)
  Please specify other reason: ____

Have you experienced any of the following side effects related to your cannabis use? If so, please indicate the severity of those side effects. If you have not experienced this side effect from cannabis use, please select I "Do not experience this side effect."

	Do not experience this side effect	1 (mild)	2	3	4 (moderate)	5	6	7 (severe)
Anxiety	◯	◯	◯	◯	◯	◯	◯	◯
Mood Issues	◯	◯	◯	◯	◯	◯	◯	◯
Dizziness	◯	◯	◯	◯	◯	◯	◯	◯
Falls	◯	◯	◯	◯	◯	◯	◯	◯
Nausea	◯	◯	◯	◯	◯	◯	◯	◯
Confusion	◯	◯	◯	◯	◯	◯	◯	◯
Other	◯	◯	◯	◯	◯	◯	◯	◯

Please specify:

____

Have you ever stopped using cannabis as a result of any of these experiences?

◯ Yes
◯ No

Symptom Improvement

These next few questions ask about cancer symptoms or cancer treatment side effects, and how your use of cannabis has affected them. If you do not experience these symptoms, please select "I do not have this symptom". For each of the following symptoms, how much has cannabis worsened or improved it? If you do not experience these symptoms, please select "I do not have this symptom."

	Worsened quite a bit	Somewhat worsened	No change	Somewhat improved	Improved quite a bit	I do not have this symptom
Pain	○	○	○	○	○	○
Mood changes, stress, anxiety, or depression	○	○	○	○	○	○
Neuropathy (numbness or tingling in your hands or feet)	○	○	○	○	○	○
Insomnia or difficulty sleeping	○	○	○	○	○	○
Loss of appetite	○	○	○	○	○	○
Digestive problems (nausea, vomiting, diarrhea, constipation)	○	○	○	○	○	○
Decline in sexual interest or activity	○	○	○	○	○	○
Sweating symptoms (e.g. hot flashes, night sweats)	○	○	○	○	○	○
Skin problems	○	○	○	○	○	○
Difficulty concentrating	○	○	○	○	○	○
Lack of energy	○	○	○	○	○	○
Other	○	○	○	○	○	○

Please specify: ____

Has cannabis worsened any of your other cancer symptoms or cancer treatment side effects?

◯ Yes
◯ No

Has cannabis improved any of your other cancer symptoms or cancer treatment side effects?

◯ Yes
◯ No

Since your cancer diagnosis, have you ever stopped using cannabis or used it less than you would like?

◯ Yes
◯ No

Did you stop using cannabis or use it less than you would like for any of the following reasons? Select all that apply.

- ☐ Cost is too high
- ☐ Not covered by my health insurance
- ☐ My health care providers have not suggested or recommended it
- ☐ My healthcare providers have recommended against it
- ☐ It did not help my symptoms
- ☐ I did not know how to get it
- ☐ Too many choices or unsure which products are safe and effective
- ☐ Concern about interaction with other medicines
- ☐ Concern about side effects
- ☐ I had a bad experience with cannabis
- ☐ Concern about legal consequences
- ☐ Concern about job loss or negative career impacts
- ☐ Concern about negative reactions from family members or friends
- ☐ It goes against my personal beliefs
- ☐ Other (please specify)

Please specify:

Pain, Mood, Health

Over the last 2 weeks how often have you been bothered by the following problems?

	Not at all	Several Days	More than half the days	Nearly every day
Little interest or pleasure in doing things	◯	◯	◯	◯
Feeling down, depressed, or hopeless	◯	◯	◯	◯

---

	0 (no pain)	1	2	3	4	5	6	7	8	9	10 (pain as bad as you can imagine)
What number best describes your pain on average in the past week:	◯	◯	◯	◯	◯	◯	◯	◯	◯	◯	◯
What number best describes how, during the past week, pain has interfered with your enjoyment of life?	◯	◯	◯	◯	◯	◯	◯	◯	◯	◯	◯
What number best describes how, during the past week, pain has interfered with your general activity?	◯	◯	◯	◯	◯	◯	◯	◯	◯	◯	◯

In general, would you say your health is:

◯ Excellent
◯ Very Good
◯ Good
◯ Fair
◯ Poor

The next few questions ask about opioid use. When we use the term 'opioid', we are referring to medicines like morphine, oxycodone/Oxycontin, hydromorphone/Dilaudid, methadone, or Fentanyl.

Are you currently using/have you ever used opioids to manage pain?

◯ Currently using opioids
◯ Recently (within last 3 months) used opioids, but no longer using them
◯ Have used it in the past but not recently (within last 3 months)
◯ Never used opioids

Have you ever used cannabis instead of opioids to manage pain?

◯ Yes
◯ No

Why did you use cannabis instead of opioids to manage pain? (check all that apply)

☐ I feel that cannabis is safer
☐ I feel that cannabis is less addictive
☐ I feel that cannabis has fewer side effects
☐ I was able to lower my dose of opioids
☐ Other reason (specify)
Please specify:

How well do you think cannabis helped to manage your pain?

◯ Cannabis was better in managing pain than opioids
◯ Opioids were better in managing pain than cannabis
◯ Cannabis and opioids managed my pain about the same way

Demographic Information

Age (in years): ____________________

What sex were you assigned at birth, on your original birth certificate?

◯ Male
◯ Female

How do you currently describe yourself? (check one)

- ◯ Male
- ◯ Female
- ◯ Transgender
- ◯ None of the above

Which of the following best represents how you think of yourself?

- ◯ Gay or lesbian
- ◯ Straight, that is, not gay or lesbian
- ◯ Bisexual
- ◯ Something else
- ◯ I am not sure yet

What is your race? One or more categories may be selected. Mark all that apply.

- ☐ White
- ☐ Black or African American
- ☐ American Indian or Alaska Native
- ☐ Asian Indian
- ☐ Chinese
- ☐ Filipino
- ☐ Japanese
- ☐ Korean
- ☐ Vietnamese
- ☐ Other Asian
- ☐ Native Hawaiian
- ☐ Guamanian or Chamorro
- ☐ Samoan
- ☐ Other Pacific Islander
- ☐ Other (please specify)
  Please specify:

Are you Hispanic, Latino/a, or Spanish origin? Mark all that apply.

- ☐ Mexican American, Chicano/a
- ☐ Puerto Rican
- ☐ Cuban
- ☐ Other Hispanic, Latino/a, or Spanish origin
- ☐ None of these

Thinking about members of your family living in this household, what is your combined annual income, meaning the
total pre-tax income from all sources earned in the past year?

- ◯ $0 to $9,999
- ◯ $10,000 to $14,999
- ◯ $15,000 to $19,999
- ◯ $20,000 to $34,999
- ◯ $35,000 to $49,999
- ◯ $50,000 to $74,999
- ◯ $75,000 to $99,999
- ◯ $100,000 to $199,999
- ◯ $200,000 or more

Which one of these comes closest to your own feelings about your household's income these days?

- ◯ Living comfortably on present income.
- ◯ Getting by on present income.
- ◯ Finding it difficult on present income.
- ◯ Finding it very difficult on present income

Do you have any kind of health care coverage, including health insurance, prepaid plans such as HMOs, or
government plans such as Medicare, or Indian Health Service?

- ◯ Yes
- ◯ No

What is the primary source of your health care coverage?

- ◯ A plan purchased through an employer or union (including plans purchased through another person's employer)
- ◯ A plan that you or another family member buys on your own
- ◯ Medicare
- ◯ Medicaid or other state program
- ◯ TRICARE (formerly CHAMPUS), VA, or Military
- ◯ Alaska Native, Indian Health Service, Tribal Health Services
- ◯ Some other source
- ◯ None (no coverage)

What is the highest grade or level of schooling you completed?

- ◯ Less than 8 years
- ◯ 8 through 11 years
- ◯ 12 years or completed high school
- ◯ Post high school training other than college (vocational or technical)
- ◯ Some college
- ◯ College graduate
- ◯ Postgraduate

What is your marital status? Mark only one.

- ◯ Married
- ◯ Living as married
- ◯ Divorced
- ◯ Widowed
- ◯ Separated
- ◯ Single, never been married

What is your current occupational status? Mark only one.

- ◯ Employed
- ◯ Unemployed
- ◯ Homemaker
- ◯ Student
- ◯ Retired
- ◯ Disabled
- ◯ Other (Specify):

Please specify: ______________________________

Were you born in the United States?

- ◯ Yes
- ◯ No
